# Bilateral Cerebellar Hemispheric Ischemia in Cryptococcal Meningitis

**DOI:** 10.7759/cureus.93775

**Published:** 2025-10-03

**Authors:** Abdul Safi, John L Liu, Mittal Prajapati, Ram Narayan

**Affiliations:** 1 Neurology, Creighton University School of Medicine, Omaha, USA

**Keywords:** corticosteroids, cryptococcus, infarction, meningitis, vasculopathy

## Abstract

Bilateral cerebellar hemispheric ischemia is a rare complication of cryptococcal meningitis that may lead to significant neurologic morbidity. We present the case of a 70-year-old male with asplenia who developed progressive headaches, confusion, and fever. Neurological exam and brain MRI were unremarkable. Cerebrospinal fluid (CSF) showed lymphocytic pleocytosis with elevated protein and low glucose levels. CSF polymerase chain reaction (PCR) consisted of *Cryptococcus neoformans*, and the patient was started on induction therapy. On day 12, the patient reported altered mental status with worsening confusion and dizziness. MRI revealed multiple small infarcts of the posterolateral left cerebellar hemisphere. Transthoracic echocardiogram (TTE) and computed tomography angiography (CTA) of the head and neck were normal. By weeks 3 and 4, new symptoms developed, including ataxia, left-sided facial droop, diplopia, and dysphagia. Repeat MRI showed increased diffusion-positive infarcts involving both cerebellar hemispheres. Due to concern for cryptococcal vasculitis, oral prednisone was initiated. Neurological function gradually improved over the next three to four months. This case highlights vasculitis as a potential contributor to ischemia in cryptococcal meningitis.

## Introduction

*Cryptococcus neoformans* is an encapsulated yeast that can cause life-threatening meningoencephalitis, most often in immunocompromised patients such as those with HIV/AIDS, organ transplants, or hematologic malignancies. In non-HIV, non-transplant patients, predisposing conditions include chronic immune dysfunction, such as asplenia, cirrhosis, or long-standing comorbidities [1,2]. Standard presenting features include headache, fever, and altered mental status [3]. Cerebrovascular complications occur in approximately 4-13% of patients and are usually due to infectious vasculopathy involving small- or medium-sized arteries [4]. These infarcts most often affect the basal ganglia, thalamus, and cerebellum and are associated with a poor prognosis [4].

Standard management consists of an induction phase with amphotericin B plus flucytosine, followed by consolidation therapy with fluconazole [5]. However, treatment may be complicated by immune reconstitution inflammatory syndrome (IRIS), characterized by paradoxical clinical worsening despite effective antifungal therapy, due to an exaggerated inflammatory response during immune recovery [6]. In selected cases with severe inflammatory complications, corticosteroids may be used. Corticosteroids are potent anti-inflammatory drugs that can help reduce the inflammation associated with IRIS, potentially improving patient outcomes. However, their use remains controversial due to potential side effects, including hyperglycemia, secondary infections, gastrointestinal bleeding, osteoporosis, mood or psychiatric changes, and delayed wound healing. Further research is needed to establish their efficacy in this context [7].

## Case presentation

A 70-year-old male with a history of childhood splenectomy and chronic portal vein thrombosis presented with three months of progressively worsening daily headaches, waxing and waning confusion, decreased appetite, and low-grade fevers. His past medical history also included atrial fibrillation without anticoagulation and hyperlipidemia. He denied alcohol and tobacco use.

At the onset of these symptoms three months earlier, he was hospitalized with daily fevers and severe headaches. He was found to have a small, punctate infarct in the right middle cerebral artery (MCA) cortical branch territory. The lesion measured approximately 3-4 mm on MRI and was clinically silent; he had no hemiparesis, aphasia, or visual field deficits at that time. His atrial fibrillation was considered a potential embolic source; however, given the tiny infarct size, lack of recurrent episodes, and concurrent systemic infection, the etiology was felt more likely to be inflammatory or cryptogenic. He was not started on anticoagulation due to a low CHA_2_DS_2_-VASc score and perceived low risk of recurrence. He was discharged oriented and without residual neurological deficits. He also tested positive for West Nile Virus (positive IgG/negative IgM), consistent with chronic infection. At discharge, he was oriented and without significant cognitive or motor deficits.

**Figure 1:**
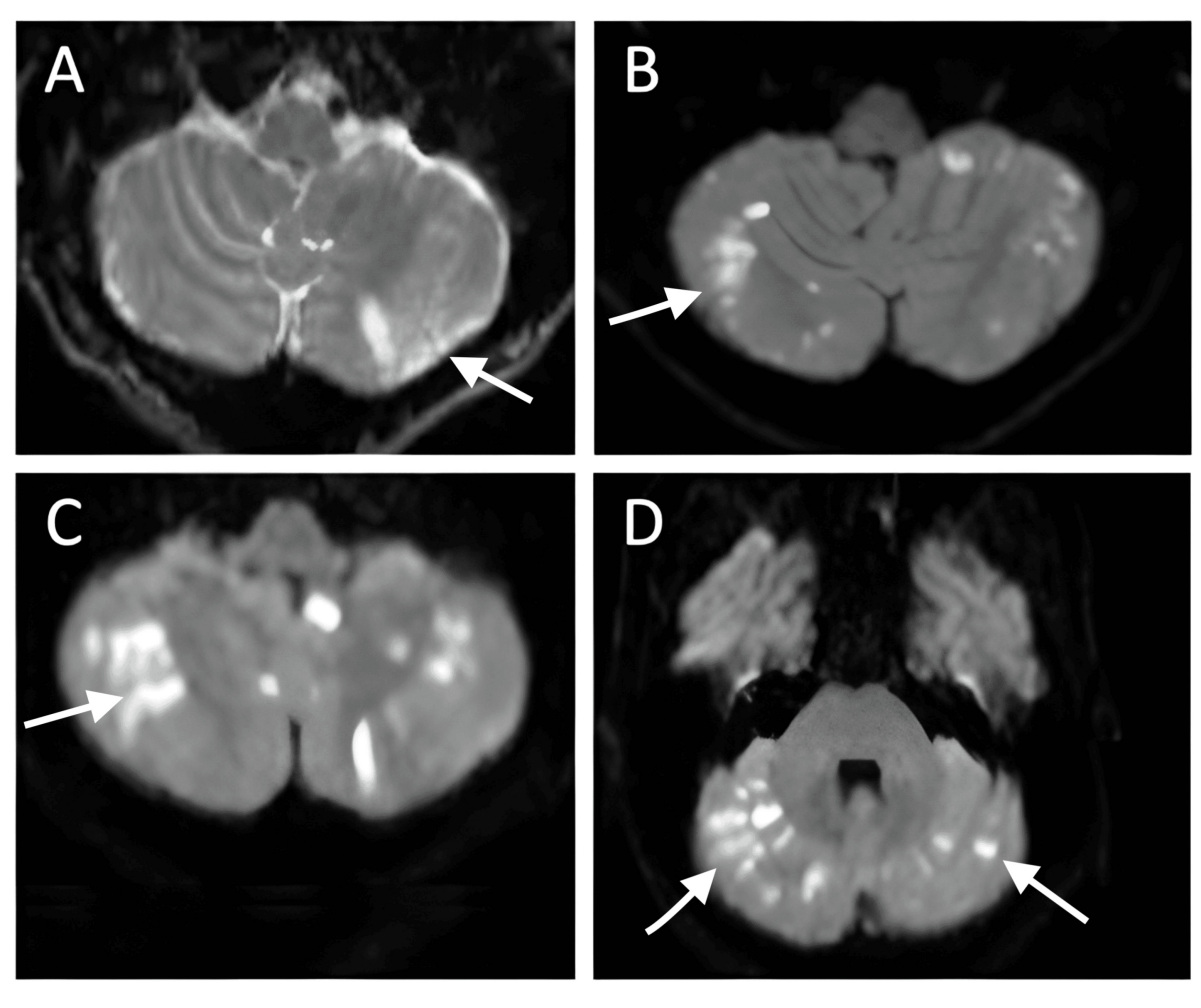
Axial diffusion weighted MRI scans in a 70-year-old male patient following the diagnosis of cryptococcal meningitis from initial presentation showing infarcts in the posterolateral cerebellar hemisphere (A). More numerous diffusion positive infarcts involving both cerebellar hemispheres are seen at 2 weeks (B), 3 weeks (C,D)

On admission, he was febrile, tachycardic, and hypertensive, but alert and oriented without focal deficits. CT of the head and cervical spine was unremarkable. Still, brain MRI demonstrated a small, focal right MCA cortical branch infarct (Figure 1A), consistent with a subacute lesion and without associated neurological deficits. Lumbar puncture showed transparent CSF with an opening pressure of 10 cm H_2_O, lymphocytic pleocytosis (54 WBC/mL, 60% lymphocytes), glucose <10 mg/dL, and protein 204 mg/dL. BioFire FilmArray (BioFire Diagnostics, Salt Lake City, UT, USA) meningitis panel was positive for *Cryptococcus neoformans *(titer 1:2560) and confirmed by CSF culture. Gram stain showed many polymorphonuclear leukocytes. Other infectious work-up, including HIV, was negative. He began induction therapy with liposomal amphotericin B and flucytosine. Lab values are indicated in Table 1.

**Table 1:** Pertinent cerebrospinal fluid and laboratory findings at presentation CSF: cerebrospinal fluid; PMN: polymorphonuclear leukocyte; ME panel: meningitis/encephalitis panel

Parameter	Patient value	Reference/normal range
CSF opening pressure	10 cm H_2_O	6–20 cm H_2_O
CSF appearance	Clear, colorless	Clear, colorless
CSF WBC count	54 /µL (60% lymphocytes)	<5 /µL
CSF glucose	<10 mg/dL	40–70 mg/dL (≈2/3 serum)
CSF protein	204 mg/dL	15–45 mg/dL
CSF cryptococcal antigen	Positive, titer 1:2560	Negative
CSF culture	Cryptococcus neoformans isolated	No growth
CSF Gram stain	Many polymorphonuclear leukocytes	No PMNs/organisms
BioFire ME panel	Positive for Cryptococcus neoformans	Negative
HIV Ag/Ab	Negative	Negative
West Nile virus serology (prior admission)	IgG positive/IgM negative	Negative

By day 12, he developed worsening confusion and dizziness. MRI revealed multiple small infarcts in the posterolateral left cerebellar hemisphere with leptomeningeal enhancement (Figure 1B). Echocardiography, CT angiogram of the brain and neck, and venous duplex studies excluded cardiac or vascular embolic sources.

After a gradual improvement in mental status and resolution of fevers, with stable vital signs and no new neurological deficits, he was discharged on day 19 to begin consolidation with fluconazole (800 mg daily for eight weeks). Thirty-six hours later, he was readmitted with new nausea, ataxia, left facial droop, diplopia, fatigue, diaphoresis, and dysphagia. He had sustained multiple falls due to an imbalance. Neurologic exam showed the patient was alert and oriented to person, place, and time. Cranial nerve exam showed left lower motor neuron facial weakness consistent with Bell’s palsy (CN VII), impaired abduction of the left eye suggestive of left CN VI palsy (correlating with pontine involvement), and absent gag reflex indicating involvement of CN IX/X. Pupils were equal, round, and reactive to light; no ptosis was noted. Extraocular movements were otherwise intact. Speech was mildly dysarthric. Motor strength was 5/5 in all extremities with no pronator drift. Sensation was intact to light touch and pinprick. Cerebellar testing revealed dysmetria on finger-to-nose on the left and a wide-based, ataxic gait with positive Romberg sign. Deep tendon reflexes were symmetric. The neck was supple but exhibited mild nuchal rigidity. MRI revealed new bilateral cerebellar infarcts and a right hippocampal infarct (not shown) (Figure 1C). Given the patient’s new neurologic deficits (ataxia, left facial droop, diplopia, and dysphagia), a repeat MRI was obtained shortly after the prior scan, which demonstrated increased diffusion-positive infarcts involving both cerebellar hemispheres (Figure 1D).

He was started on prednisone (10 mg daily, later increased to 60 mg with taper) for suspected IRIS-related vasculopathy. Trimethoprim-sulfamethoxazole (TMP-SMX) was added for *Pneumocystis* prophylaxis. Follow-up MRI showed improvement in cerebellar signal changes. His admission to rehabilitation for impaired mobility and speech was a turning point. Over the following four weeks, his cognition and mobility improved significantly, instilling hope for his eventual discharge home.

## Discussion

This patient developed multifocal ischemic infarcts during treatment for cryptococcal meningitis, likely secondary to infectious vasculopathy. While cerebral infarctions are an uncommon complication, they are well described and may result from fungal invasion, vasculitis, or thrombotic occlusion [2]. In this case, involvement of both cerebellar hemispheres and the hippocampus was less common than the typical basal ganglia or thalamic distribution [2].

Negative vascular imaging and echocardiography, along with the infarct pattern, suggested an inflammatory rather than embolic mechanism. Continued progression despite antifungal therapy raised concern for IRIS, previously described in both HIV-positive and HIV-negative patients [3].

Adjunctive corticosteroids 

In cryptococcal meningitis, corticosteroids are usually avoided early but may be indicated for: (1) new or worsening neurologic deficits with progression on imaging despite antifungal therapy; (2) evidence of IRIS or post-infectious inflammatory response syndrome (PIIRS) with negative cultures; or (3) severe CNS inflammation with mass effect or intracranial hypertension. Guidelines recommend prednisone-equivalent 0.5-1.0 mg/kg/day for two to six weeks with taper [1,4].

Host factors and risk 

The patient's asplenia reflects chronic immune dysfunction, impairing opsonization and increasing fungal burden. Chronic portal vein thrombosis, while not a known risk factor, may have compounded ischemic risk in the setting of vasculitis. Other comorbidities such as cirrhosis, malignancy, or prior glucocorticoid exposure are associated with worse outcomes [1,4].

Long-term outcomes

Stroke complicating cryptococcal meningitis is linked with poor prognosis. In HIV-negative cohorts, infarctions increase the odds of poor outcomes more than twelvefold [5]. Survivors frequently suffer cognitive impairment, cranial neuropathies, or motor deficits, with 20-70% disability at follow-up [6]. Functional recovery may peak at 1 month but improve slowly with structured rehabilitation [6].

This patient improved after corticosteroid escalation, supporting a role for steroids in select cases. TMP-SMX was used to mitigate infection risk during prolonged immunosuppression.

## Conclusions

Vasculopathic complications in cryptococcal meningitis are rare but may cause recurrent infarctions and disability. In patients with neurologic deterioration despite antifungal therapy and negative CSF cultures, IRIS or post-infectious inflammatory response syndrome (PIIRS) should be considered. A time-limited corticosteroid course can be practical in carefully selected cases. Early recognition, repeat imaging, and multidisciplinary management are critical to optimizing outcomes.
